# Evaluation of skeletal muscle activity during foot training exercises using positron emission tomography

**DOI:** 10.1038/s41598-022-11202-y

**Published:** 2022-04-30

**Authors:** Tomoyuki Kanayama, Junsuke Nakase, Takafumi Mochizuki, Kazuki Asai, Rikuto Yoshimizu, Mitsuhiro Kimura, Seigo Kinuya, Hiroyuki Tsuchiya

**Affiliations:** 1 Department of Orthopedic Surgery, Graduate School of Medical Sciences, Kanazawa University, 13-1 Takara-machi, Kanazawa-city, 920-8641 Japan; 2 Kanazawa Advanced Medical Center, Kanazawa, Japan; 3 Department of Nuclear Medicine/Biotracer Medicine, Graduate School of Medical Science, Kanazawa University, Kanazawa, Japan

**Keywords:** Medical research, Outcomes research

## Abstract

The foot exercises “rock-paper-scissors” and “towel gathering” are widely used in patients with lower limb disorders; however, there are no detailed reports on muscle activity during such training. We quantitatively evaluated the difference in skeletal muscle activity between the two exercises using positron emission tomography. Eight university student athletes were included. Four participants each were assigned to the foot rock-paper-scissors and towel gathering groups. Participants in each group underwent continuous training for 15 min, and received an intravenous injection of 18F-fluorodeoxyglucose. After retraining for 15 min, participants rested for 45 min. Regions of interest were defined in 25 muscles. The standardized uptake value (SUV) in the trained limb was compared with that in the non-trained control limb. SUVs increased in four skeletal muscles (tibialis anterior, peroneus brevis, extensor hallucis brevis, and abductor hallucis) in the rock-paper-scissors group, and in four muscles (flexor digitorum longus, extensor hallucis brevis, extensor digitorum brevis, and quadratus plantae) in the towel gathering group. Thus, foot rock-paper-scissors and towel gathering involved skeletal muscles related to the medial longitudinal arch and toe grip strength, respectively. Given that the two exercises target different skeletal muscles, they should be taught and implemented according to their respective purposes.

## Introduction

The foot is a complex structure consisting of 26 bones, 20 intrinsic and nine extrinsic muscles, 108 ligaments, and more than 30 joints. All these structures act in unison and enable bipedal walking^1^. When these intrinsic foot muscles weakness, these roles are impaired^2^, increasing the load on other passive foot structures and leading to foot deformities and injuries^2,3^.

**Figure 1:**
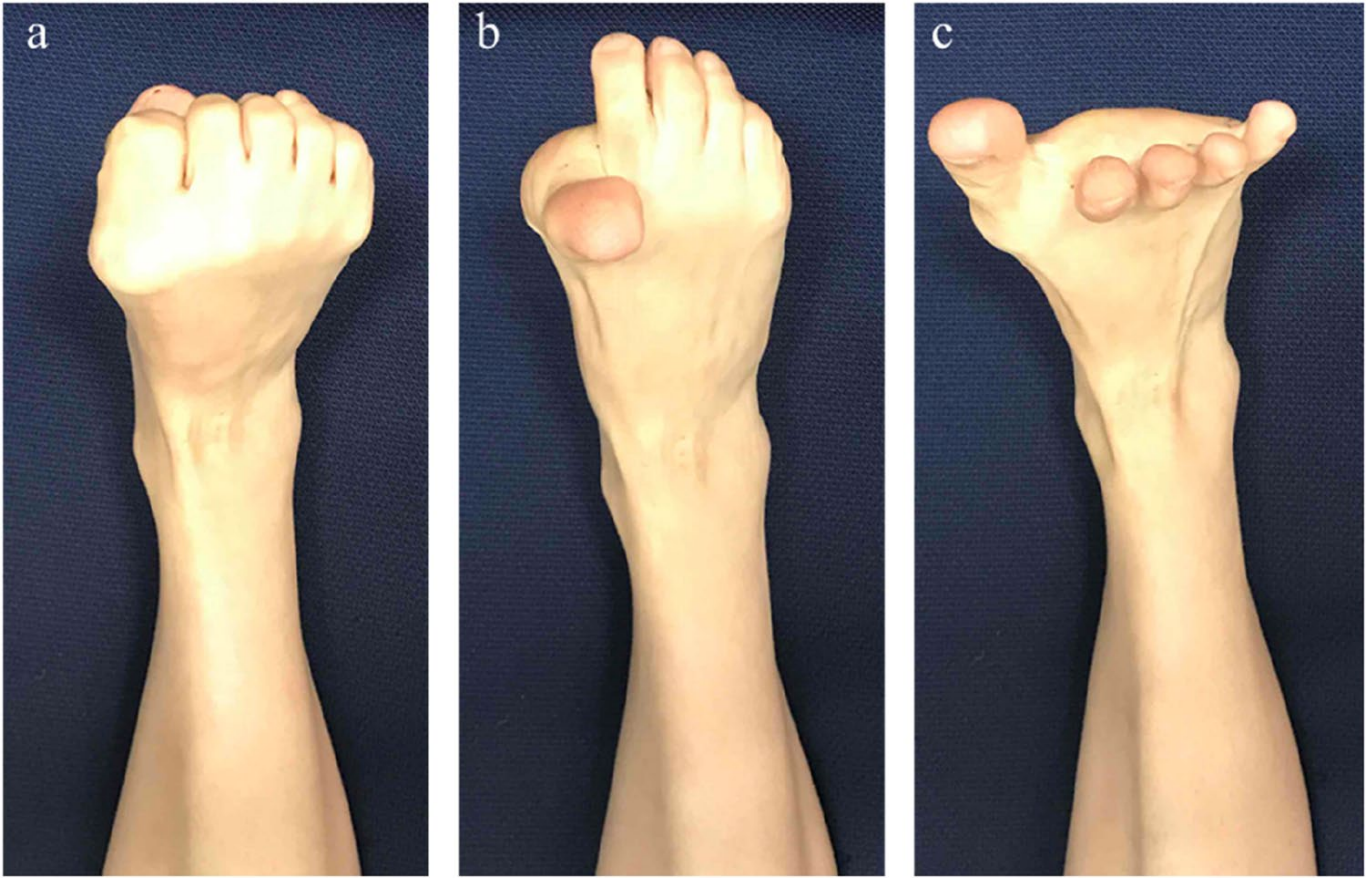
The foot rock-paper-scissors exercise. Repetition of the following movement sequence: fold the toes (**a**), spread the toes out (**b**), and extend the first toe (**c**).

Ihara et al.^4^ first reported the effectiveness of dynamic joint control training for lower limb disorders. In Japan, the foot exercises “rock-paper-scissors” and “towel gathering” are commonly used for dynamic joint control training during non-weight-bearing periods. The rock-paper-scissors exercise for the foot involves folding the toes, spreading the toes out, and extending the first toe (Fig. 1). An electromyography (EMG) study reported that the act of spreading-out the toes required activation of the abductor hallucis muscle^5,6^, while that of extending the first toe required activation of the flexor digitorum longus muscle^7^. These exercises are known to train the medial longitudinal arch of the foot and can improve dynamic balance^8^. In a magnetic resonance imaging (MRI)-based study, Goodling et al.^9^ reported that the spreading out of toes especiallyactivated the dorsal interossei, lumbricals, and abductor digiti minimi muscles, and extension of the first toe significantly activated the flexor hallucis brevis and the flexor digitorum brevis muscles.

**Figure 2:**
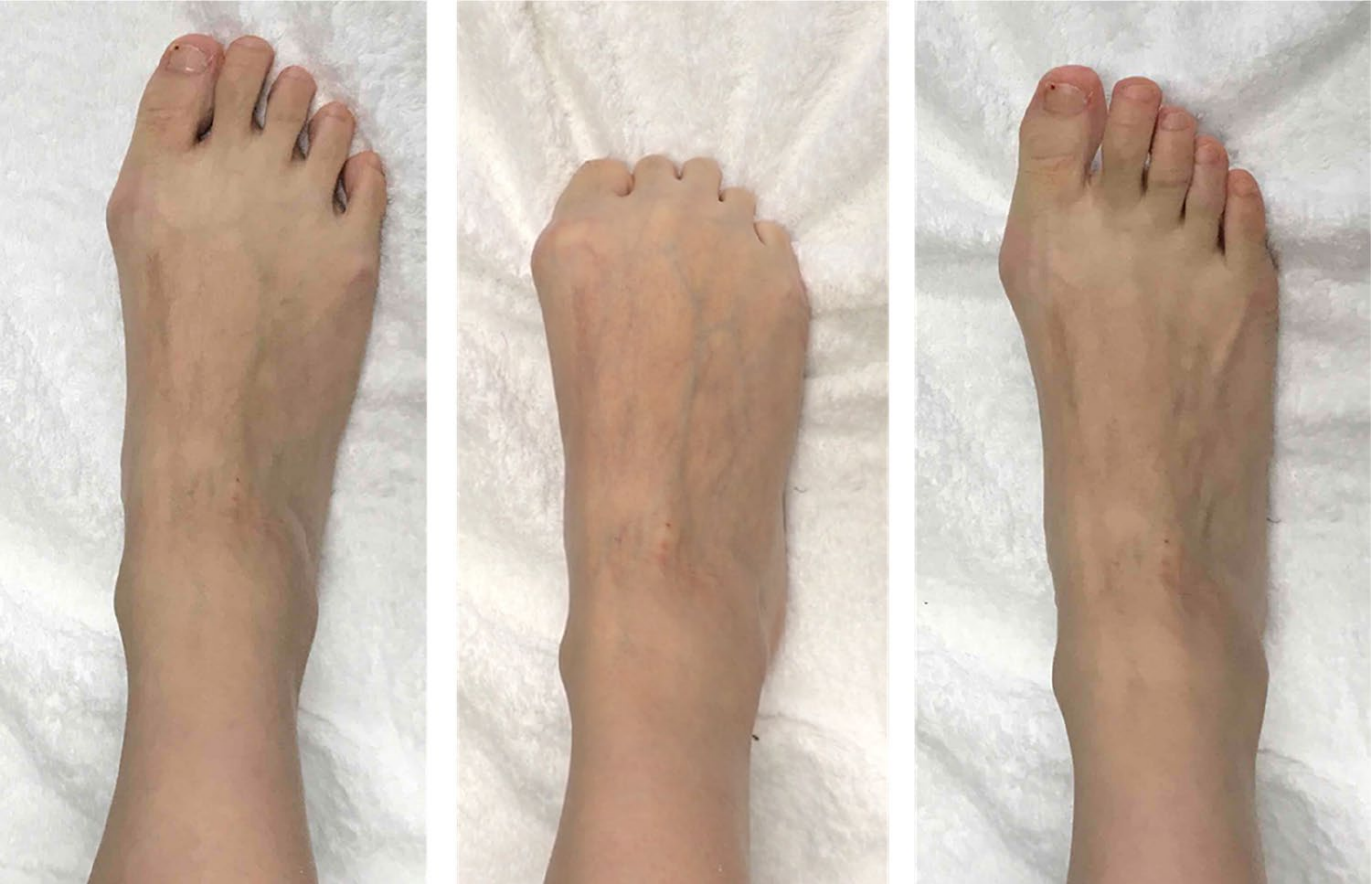
The towel-gathering exercise. With the heels on the floor, grasp the towel with the toes and repeat the movement of pulling it toward oneself and releasing it.

Towel gathering refers to the action of using one’s toes to pull a towel towards the foot (Fig. 2). While no studies have investigated skeletal muscle activity during towel gathering, some have examined a similar exercise known as the “toe curl.” As toe curls strengthen the flexor digitorum longus, brevis, lumbricals, and flexor hallucis longus, they are thought to be useful mainly for flexion of the toes^10^. The medial longitudinal arch is supported by the flexor hallucis longus, flexor digitorum longus, abductor hallucis, flexor digitorum brevis, and tibialis posterior muscles^11^. When the medial longitudinal arch of the foot is decreased, the load is not distributed correctly, resulting in worsenedbalance^8,12^.

EMG, which detects electric potentials caused by transmembrane currents in muscle fibers, has been used to obtain electrophysiological recordings of muscle activity during training^5–10^. Such recordings make it possible to compare skeletal muscle activity during different exercises^13^. However, EMG has some limitations. In general, the use of surface electrodes allows for recording from a limited number of superficial muscles. Though needle electrodes can be used to observe deeper muscles, they are somewhat invasive. In addition, the cables connected to the electrodes interfere with exercise, disrupting the activity level and limiting the types of exercise that can be evaluated.

Glucose metabolism during exercise is dependent on the power output of the muscle and the recruited muscle mass; thus, the tissue uptake of plasma glucose increases with increasing exercise intensity^14,15^. Fujimoto et al.^16,17^ focused on the mechanism of glucose metabolism in skeletal muscle using positron emission tomography (PET) with ^18^F-fluorodeoxyglucose (FDG), reporting levels of glucose uptake in individual skeletal muscles during aerobic exercise. Subsequent studies have shown that glucose metabolism measured by FDG-PET is highly correlated with the intensity of skeletal muscle activity^18,19^.

However, few studies have examined skeletal muscle activity during foot training exercises. Among them, most EMG studies have focused on the flexor muscle group^5–8,10^, while MRI studies have examined the area distal to the ankle joint^9^. No study has quantitatively evaluated the skeletal muscle activity of the whole lower limb during each exercise.

In the present study, we aimed to quantitatively evaluate the skeletal muscle activity during each foot training exercise using PET. We hypothesized that (i) the activity of the muscle groups involved in the medial longitudinal arch would increase during the rock-paper-scissors exercise, and that (ii) the activity of the muscle groups involved in toe grip strength, especially that of the flexor group, would increase in the towel gathering exercise.

## Materials and methods

This research complies with the principles of the Helsinki Declaration. The study design was approved by the Kanazawa University Clinical Trials Ethics Review Committee (approval #6132). The purpose and potential risks of this study were explained to all the participants, following which they provided written informed consent. We obtained approval of the study protocol and informed the participants prior to the trial start date. We prospectively registered the trial in the Infrastructure for Academic Activities University Hospital Medical Information Network (UMIN000044554). We performed this study at the Kanazawa Advanced Medical Center, Kanazawa, Japan. All participants were recruited in June 2021, and all trials were performed in June 2021.

Eight healthy university athletes participated in the study after four declined to participate. Four participants were randomly assigned (allocation ratio 1:1) to the rock-paper-scissors (RPS) group, while the remaining four were assigned to the towel gathering (TG) group. For each group, the right foot was assigned for training, while the left foot took no part in the exercise and was designated as the control. All participants were considered healthy after a careful review of their medical history and physical examination.

All participants refrained from strenuous exercise the day before the test and were not allowed to eat or drink anything except water for 6 h prior to the test, to allow for maximum glucose uptake and utilization by the muscles. The participants in each group underwent continuous training for 15 min. They received an intravenous injection of 37 MBq of FDG and retrained for 15 min, following which they remained seated and at rest for 45 min. Voluntary muscles that actively contracted during the FDG uptake phase (primarily 30 min after tracer administration) would have had increased FDG accumulation. FDG-PET is usually performed at least 50 min after intravenous administration of FDG. This interval allows for enhanced tracer activity due to the intracellular capture of FDG (as FDG6-phosphate). Concomitantly, the pooling of the tracer in the blood is decreased, and the overall background tracer activity is lowered, thereby improving the background ratio. Although the background ratio can be improved even after 1 h post-injection, most facilities start emission image acquisition at about 1 h-mark post-injection, because of the reduction in counting statistics given the short physical half-life of 18F (110 min)^20^. In the present study, following a previous report by our group^21^, imaging was performed at 60 min after the injection. Before FDG injection, plasma glucose levels were confirmed to be normal, and PET-computed tomography (CT) was performed. During the training session, an orthopedic surgeon with more than 6 years of experience monitored participants to ensure that there were no problems with their exercise regimen.

## PET analysis

Following the post-training 45-min rest period, the participants lied in a supine position in the gantry of the PET-CT system (Discovery PET/CT 690; GE Healthcare, Milwaukee, WI, USA). The scan was performed with a 60-cm axial field-of-view and a transaxial resolution of 6.4 mm (full-width at half maximum at the center of the field-of-view without a scattering medium). Prior to emission scanning, an unenhanced CT scan was performed for attenuation correction and muscle orientation. Emission scanning was performed in the three-dimensional mode, 60 min after 18F-FDG administration at 3 min/bed station. The total emission time was 15–21 min. The three-dimensional ordered subset expectation maximization method was used to reconstruct the images, with two iterations and 16 subsets. A 6.4-mm full-width at half-maximum Gaussian post-filter was applied after reconstruction.

Twenty-five skeletal muscles identified by plain CT axial imaging were used for the evaluation. The gastrocnemius muscle was evaluated in two parts, one each for the medial and lateral heads. When evaluating each skeletal muscle, landmarks were stipulated to minimize the deviation of the slices to be evaluated. As in previous reports^21,26^, SUV values were measured in the slice with the maximum skeletal muscle belly. The combinations of the stipulated landmarks and the 25 skeletal muscles evaluated were as follows: tibialis anterior, extensor hallucis longus, extensor digitorum longus, peroneus tertius, peroneus longus, peroneus brevis, gastrocnemius, soleus, plantaris, popliteus, flexor digitorum longus, flexor hallucis longus, tibialis posterior, extensor hallucis brevis, extensor digitorum brevis, abductor hallucis, flexor hallucis brevis, flexor digitorum brevis, adductor hallucis, abductor digiti minimi, flexor digiti minimi brevis, opponens digiti minimi, quadratus plantae, lumbrical, and interosseous.

Regions of interest (ROIs) were manually drawn for the 25 skeletal muscles. An experienced orthopedic surgeon defined all ROIs using plain CT images and calculated the standardized uptake value (SUV) for FDG. The SUV was calculated according to the following equation to quantify the FDG uptake of the muscle tissue per unit volume:

$$\mathrm{SUV }=\frac{\mathrm{mean\,region\,of\,interest\,count }\times \mathrm{ calibration\,factor }}{\frac{\mathrm{injected\,dose}}{\mathrm{body\,weight}}}$$

ROIs were defined on the right and left sides of the skeletal muscles, as described above. The mean SUV was calculated using the following equation:

$$\mathrm{mean\,SUV}=\frac{\mathrm{left\,}(\mathrm{mean\,SUV }\times \mathrm{ muscle\,area})+\mathrm{ right }\,(\mathrm{mean\,SUV }\times \mathrm{ muscle\,area})}{\mathrm{left\,muscle\,area }+\mathrm{ right\,muscle\,area}}$$

## Sample size calculation and statistical analysis

Sample size was calculated using G-power 3.1 (effect size: 1.6, α-error: 0.05, and target power: 0.95). A minimum of four participants per group was recommended based on a previous study^22^, and four participants were thus enrolled in each group to account for unexpected injuries and withdrawal of consent.

All data are presented as the mean and standard deviation. All statistical analyses were performed using IBM SPSS for Windows ver. 25.0 (IBM Corp., Armonk, NY, USA). The independent-samples t-test was used to evaluate differences in the mean SUV between the RPS training and TG training groups and the RPS control and TG control groups. The differences between the SUVs of the RPS training and RPS control groups and those between the TG training and TG control groups were evaluated using a paired-samples t-test. Statistical significance was set at P < 0.05.

### Ethical approval

This research complies with the principles of the Helsinki Declaration. The study title was as follows: “Examination of the effect of foot training using positron emission tomography (PET) on the intrinsic muscles of the foot -to update the anterior cruciate ligament injury preventive program.” The study design was approved by the Kanazawa University Clinical Trials Ethics Review Committee (approval #6132). The purpose and potential risks of this study were explained to the participants, and written informed consent was obtained from all participants. We obtained approved the protocol of the study and provided information to participants before the trial start date. We prospectively registered the trial in the Infrastructure for Academic Activities University Hospital Medical Information Network (UMIN000044554).

### Consent for publication

The research participation agreement form also included an item of public consent.

## Results

There were no significant differences in baseline characteristics (age, height, body weight, and Body mass index) between the RPS and TG groups (Table 1).

**Table 1:** Physical characteristics of participants in the RPS and TG groups.

	RPS group	TG group	p-value
No. of participants	4	4	
Age, years	20.5 ± 0.6	20.8 ± 1.0	0.67
Height, cm	173.1 ± 10.3	176.4 ± 10.4	0.67
Weight, kg	73.5 ± 12.7	79.7 ± 6.9	0.42
Body mass index, kg/m^2^	24.5 ± 3.8	25.7 ± 2.1	0.61

Values are presented as the mean ± standard deviation.

RPS: rock-paper-scissors; TG: towel gathering. The independent-samples t-test was used to evaluate differences between the RPS and TG groups. Statistical significance was set at P < 0.05.

**Figure 3:**
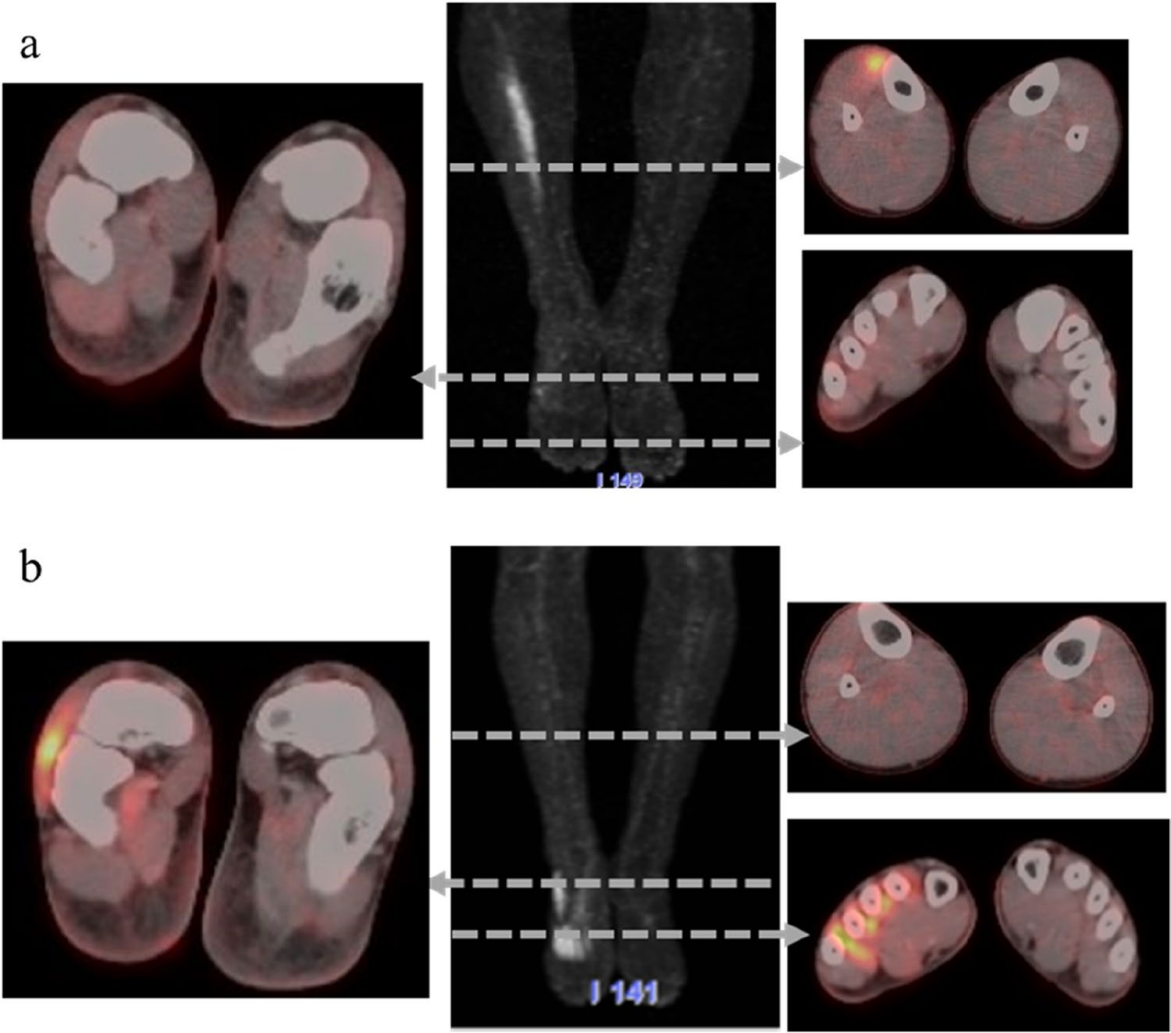
Representative lower-body positron emission tomography images after exercise performance. Positron emission tomography image and axial section of fusion computerized tomography from the foot rock-paper-scissors group (**a**) and the towel-gathering group (**b**).

Typical lower-body PET images of the RPS and TG groups are shown in Fig. 3. In the RPS group, four muscles (flexor digitorum longus, extensor hallucis brevis, extensor digitorum brevis, and quadratus plantae) exhibited a significant increase in the SUV in the training limb compared with that in the control limb (Table 2). In the TG group, four other muscles (tibialis anterior, peroneus brevis, extensor hallucis brevis, and abductor hallucis) exhibited a significant increase in the SUV in the training limb compared with that in the control limb (Table 3). For all skeletal muscles, there were no differences between SUVs in the RPS control and TG control groups (Table 4). Only the SUV of the extensor hallucis longus muscle exhibited a significant difference between the RPS training and TG training groups (Table 5).

**Table 2:** Mean SUVs in the RPS training and control groups.

Muscles	Mean SUVs		p-value
	RPS training group	RPS control group	
Tibialis anterior muscle	2.26 ± 1.74	0.87 ± 0.44	0.125
Extensor hallucis longus muscle	1.96 ± 1.00	0.82 ± 0.27	0.076
Extensor digitorum longus muscle	0.89 ± 0.21	0.63 ± 0.10	0.100
Peroneus tertius muscle	0.73 ± 0.21	0.68 ± 0.26	0.395
Peroneus longus muscle	0.76 ± 0.18	0.60 ± 0.03	0.155
Peroneus brevis muscle	0.82 ± 0.18	0.54 ± 0.10	0.056
Medial head of gastrocnemius muscle	0.62 ± 0.11	0.60 ± 0.14	0.471
Lateral head of gastrocnemius muscle	0.64 ± 0.13	0.58 ± 0.09	0.111
Soleus muscle	0.74 ± 0.15	0.73 ± 0.14	0.565
Plantaris muscle	0.61 ± 0.09	0.53 ± 0.13	0.150
Popliteus muscle	0.98 ± 0.51	0.69 ± 0.13	0.228
Flexor digitorum longus muscle	0.66 ± 0.11	0.60 ± 0.13	**0.012**
Flexor hallucis longus muscle	0.74 ± 0.15	0.58 ± 0.13	0.055
Tibialis posterior muscle	0.76 ± 0.21	0.64 ± 0.12	0.147
Extensor hallucis brevis muscle	0.96 ± 0.34	0.65 ± 0.22	**0.022**
Extensor digitorum brevis muscle	1.77 ± 0.9	0.60 ± 0.18	**0.049**
Abductor hallucis muscle	0.75 ± 0.32	0.73 ± 0.22	0.855
Flexor hallucis brevis muscle	0.88 ± 0.38	0.59 ± 0.23	0.287
Flexor digitorum brevis muscle	1.00 ± 0.40	0.68 ± 0.24	0.069
Adductor hallucis muscle	1.91 ± 1.53	0.74 ± 0.15	0.204
Abductor digiti minimi muscle	1.94 ± 1.80	0.64 ± 0.18	0.231
Flexor digiti minimi brevis muscle	1.92 ± 1.60	0.78 ± 0.19	0.208
Opponens digiti minimi muscle	0.97 ± 0.37	0.72 ± 0.17	0.090
Quadratus plantae muscle	0.99 ± 0.32	0.71 ± 0.18	**0.033**
Lumbrical muscle	1.23 ± 0.80	0.64 ± 0.13	0.200
Interosseous muscle	1.3 ± 0.65	0.58 ± 0.16	0.072

Values are presented as the mean ± standard deviation.

RPS: rock-paper-scissors; SUV: standardized uptake value. The paired-samples t-test was used to evaluate differences in the mean SUV between the RPS training and control groups. Statistical significance was set at P < 0.05.

**Table 3:** Mean SUVs in the TG training and TG control groups.

Muscles	Mean SUVs		p-value
	TG training group	TG control group	
Tibialis anterior muscle	0.79 ± 0.03	0.64 ± 0.13	**0.026**
Extensor hallucis longus muscle	0.70 ± 0.15	0.61 ± 0.12	0.413
Extensor digitorum longus muscle	0.72 ± 0.11	0.55 ± 0.04	**0.048**
Peroneus tertius muscle	0.62 ± 0.10	0.52 ± 0.06	0.050
Peroneus longus muscle	0.62 ± 0.05	0.52 ± 0.06	0.127
Peroneus brevis muscle	0.72 ± 0.05	0.57 ± 0.08	0.016
Medial head of gastrocnemius muscle	0.56 ± 0.07	0.54 ± 0.05	0.289
Lateral head of gastrocnemius muscle	0.56 ± 0.06	0.51 ± 0.01	0.172
Soleus muscle	0.78 ± 0.13	0.63 ± 0.04	0.054
Plantaris muscle	0.63 ± 0.16	0.53 ± 0.10	0.160
Popliteus muscle	0.73 ± 0.53	0.70 ± 0.03	0.486
Flexor digitorum longus muscle	0.73 ± 0.09	0.63 ± 0.07	0.210
Flexor hallucis longus muscle	1.07 ± 0.59	0.60 ± 0.07	0.243
Tibialis posterior muscle	0.92 ± 0.21	0.68 ± 0.15	0.234
Extensor hallucis brevis muscle	0.94 ± 0.11	0.79 ± 0.08	**0.042**
Extensor digitorum brevis muscle	1.28 ± 0.53	0.79 ± 0.08	0.139
Abductor hallucis muscle	0.94 ± 0.12	0.68 ± 0.09	**0.007**
Flexor hallucis brevis muscle	1.13 ± 0.57	0.64 ± 0.23	0.120
Flexor digitorum brevis muscle	1.41 ± 0.65	0.70 ± 0.12	0.124
Adductor hallucis muscle	1.03 ± 0.54	0.64 ± 0.09	0.232
Abductor digiti minimi muscle	1.78 ± 1.51	0.60 ± 0.10	0.222
Flexor digiti minimi brevis muscle	1.56 ± 0.88	0.70 ± 0.20	0.151
Opponens digiti minimi muscle	1.64 ± 1.47	0.68 ± 0.07	0.274
Quadratus plantae muscle	1.92 ± 1.44	0.61 ± 0.08	0.168
Lumbrical muscle	1.15 ± 0.29	0.89 ± 0.12	0.151
Interosseous muscle	2.18 ± 1.69	0.58 ± 0.14	0.160

Values are presented as the mean ± standard deviation. TG: towel gathering; SUV: standardized uptake value. The paired-samples t-test was used to evaluate differences in the mean SUV between the TG training and control groups. Statistical significance was set at P < 0.05.

**Table 4:** Comparison of mean SUVs in the RPS control and TG control groups.

Muscles	Mean SUVs		p-value
	RPS control group	TG control group	
Tibialis anterior muscle	0.87 ± 0.44	0.64 ± 0.13	0.370
Extensor hallucis longus muscle	0.82 ± 0.27	0.61 ± 0.12	0.203
Extensor digitorum longus muscle	0.63 ± 0.10	0.55 ± 0.04	0.201
Peroneus tertius muscle	0.68 ± 0.26	0.52 ± 0.06	0.260
Peroneus longus muscle	0.60 ± 0.03	0.52 ± 0.06	0.056
Peroneus brevis muscle	0.54 ± 0.10	0.57 ± 0.08	0.667
Medial head of gastrocnemius muscle	0.60 ± 0.14	0.54 ± 0.05	0.456
Lateral head of gastrocnemius muscle	0.58 ± 0.09	0.51 ± 0.01	0.176
Soleus muscle	0.73 ± 0.14	0.63 ± 0.04	0.206
Plantaris muscle	0.53 ± 0.13	0.53 ± 0.10	0.984
Popliteus muscle	0.69 ± 0.13	0.70 ± 0.03	0.836
Flexor digitorum longus muscle	0.60 ± 0.13	0.63 ± 0.07	0.616
Flexor hallucis longus muscle	0.58 ± 0.13	0.60 ± 0.07	0.717
Tibialis posterior muscle	0.64 ± 0.12	0.68 ± 0.15	0.680
Extensor hallucis brevis muscle	0.65 ± 0.22	0.79 ± 0.08	0.262
Extensor digitorum brevis muscle	0.60 ± 0.18	0.79 ± 0.08	0.112
Abductor hallucis muscle	0.73 ± 0.22	0.68 ± 0.09	0.646
Flexor hallucis brevis muscle	0.59 ± 0.23	0.64 ± 0.23	0.761
Flexor digitorum brevis muscle	0.68 ± 0.24	0.70 ± 0.12	0.889
Adductor hallucis muscle	0.74 ± 0.15	0.64 ± 0.09	0.299
Abductor digiti minimi muscle	0.64 ± 0.18	0.60 ± 0.10	0.712
Flexor digiti minimi brevis muscle	0.78 ± 0.19	0.70 ± 0.20	0.572
Opponens digiti minimi muscle	0.72 ± 0.17	0.68 ± 0.07	0.669
Quadratus plantae muscle	0.71 ± 0.18	0.61 ± 0.08	0.353
Lumbrical muscle	0.64 ± 0.13	0.89 ± 0.12	0.051
Interosseous muscle	0.58 ± 0.16	0.58 ± 0.14	0.966

Values are presented as the mean ± standard deviation.

RPS: rock-paper-scissors; TG: towel gathering; SUV: standardized uptake value. The paired-samples t-test was used to evaluate differences in the mean SUV between the RPS control and TG control groups. Statistical significance was set at P < 0.05.

**Table 5:** Differences in mean SUVs between the RPS training and TG training groups.

Muscles	Mean SUVs		p-value
	RPS training group	TG training group	
Tibialis anterior muscle	2.26 ± 1.74	0.79 ± 0.03	0.145
Extensor hallucis longus muscle	1.96 ± 1.00	0.70 ± 0.15	**0.046**
Extensor digitorum longus muscle	0.89 ± 0.21	0.72 ± 0.11	0.214
Peroneus tertius muscle	0.73 ± 0.21	0.62 ± 0.10	0.378
Peroneus longus muscle	0.76 ± 0.18	0.62 ± 0.05	0.170
Peroneus brevis muscle	0.82 ± 0.18	0.72 ± 0.05	0.345
Medial head of gastrocnemius muscle	0.62 ± 0.11	0.56 ± 0.07	0.376
Lateral head of gastrocnemius muscle	0.64 ± 0.13	0.56 ± 0.06	0.258
Soleus muscle	0.74 ± 0.15	0.78 ± 0.13	0.714
Plantaris muscle	0.61 ± 0.09	0.63 ± 0.16	0.822
Popliteus muscle	0.98 ± 0.51	0.73 ± 0.53	0.358
Flexor digitorum longus muscle	0.66 ± 0.11	0.73 ± 0.09	0.390
Flexor hallucis longus muscle	0.74 ± 0.15	1.07 ± 0.59	0.321
Tibialis posterior muscle	0.76 ± 0.21	0.92 ± 0.21	0.330
Extensor hallucis brevis muscle	0.96 ± 0.34	0.94 ± 0.11	0.932
Extensor digitorum brevis muscle	1.77 ± 0.9	1.28 ± 0.53	0.382
Abductor hallucis muscle	0.75 ± 0.32	0.94 ± 0.12	0.312
Flexor hallucis brevis muscle	0.88 ± 0.38	1.13 ± 0.57	0.495
Flexor digitorum brevis muscle	1.00 ± 0.40	1.41 ± 0.65	0.332
Adductor hallucis muscle	1.91 ± 1.53	1.03 ± 0.54	0.318
Abductor digiti minimi muscle	1.94 ± 1.80	1.78 ± 1.51	0.881
Flexor digiti minimi brevis muscle	1.92 ± 1.60	1.56 ± 0.88	0.707
Opponens digiti minimi muscle	0.97 ± 0.37	1.64 ± 1.47	0.413
Quadratus plantae muscle	0.99 ± 0.32	1.92 ± 1.44	0.250
Lumbrical muscle	1.23 ± 0.80	1.15 ± 0.29	0.868
Interosseous muscle	1.3 ± 0.65	2.18 ± 1.69	0.371

Values are presented as the mean ± standard deviation.

RPS: rock-paper-scissors; TG: towel gathering; SUV: standardized uptake value. The paired-samples t-test was used to evaluate differences in the mean SUV between the RPS training and TG training groups. Statistical significance was set at P < 0.05.

## Discussion

This is the first study to apply FDG-PET to investigate comprehensive lower-body skeletal muscle activity during intrinsic foot muscle training. The authors attempted a detailed investigation of the contribution of each muscle of the lower extremity, and collected precise measurements to visualize relatively complex tasks of the lower extremity. The most important findings of this study were that the rock-paper-scissors exercise involves the medial longitudinal arch of the foot, while the towel-gathering exercise involves the muscles involved in toe-grip. As these findings indicate that the two exercises target different skeletal muscles, it is necessary to incorporate them into training according to their respective purposes.

Glucose is one of the energy sources for skeletal muscle. Like glucose, 18F-FDG is taken up by myocytes, and since it is not metabolized, it remains in the myocytes as FDG-6-phosphate (“metabolic trapping”)^16,17,19^. Since metabolic trapping is maintained for approximately 2 h after injection^23^, FDG-PET reflects skeletal muscle glucose metabolism during exercise. Fujimoto et al. used PET to evaluate muscle activity during exercise in one of the first PET-based studies on muscle activity during running^16^. Other studies have investigated PET during more complex tasks requiring endurance, such as running^24^ and double-poling^25^. In a previous study, our group applied FDG-PET to the FIFA 11 + training program and reported on changes in muscle activity during training^22,26^. We have also evaluated muscle activity in the lower limb using a belt-electrode skeletal muscle electrical stimulation system to demonstrate the effectiveness of FDG-PET in passive exercise^21^. These findings provide a rationale for assessing skeletal muscle activity using FDG-PET.

For isometric strengthening of the intrinsic foot muscle, the most recognized exercise is the short foot exercise^27^, in where volitional control of the intrinsic foot muscles elevates the foot arches and shortens the foot. This exercise is described as part of the core paradigm introduced by McKeon et al.^27^. The short foot exercise is typically challenging to teach and learn; therefore, three gradual training steps have been recommended. Meanwhile, foot rock-paper-scissors is a complex exercise that consists of toe clenching, spreading the toes out, and extension of the first toe in succession, but it is easy to learn. When the toes are spread out, circumferential motion occurs at the first and fifth toes, activating muscles that extend and abduct these digits.

The results of the present study showed that, as in previous investigations^5–7,9^, foot rock-paper-scissors produced significant skeletal muscle activity in the extensor hallucis brevis, flexor digitorum longus, and quadratus plantaris muscles. During towel gathering, significant skeletal muscle activity was observed in the tibialis anterior, peroneus brevis, extensor hallucis brevis, and abductor hallucis muscles. Significantly more activity in the extensor hallucis longus muscle was observed during towel gathering than during foot rock-paper-scissors (P = 0.046). There was also a tendency for skeletal muscle activity to occur in the tibialis anterior, extensor hallucis longus, extensor digitorum brevis, flexor hallucis brevis, adductor hallucis, and abductor digiti minimi muscles during the foot rock-paper-scissors exercise. However, there was large variation among individuals, and no significant difference was observed. In the towel gathering group, skeletal muscle activity tended to occur in the flexor digitorum longus, flexor hallucis longus, abductor hallucis, flexor digitorum brevis, quadratus plantae, lumbrical, and interosseous muscles. Towel gathering effectively involved not only the flexors of the toes, but also the extensors.

During towel gathering, all toes are flexed via contraction of the flexor digitorum longus and flexor hallucis longus muscles to enable grasping the towel. Next, the tibialis posterior and tibialis anterior muscles contract by dorsiflexing the ankle joint while grasping the towel. Finally, when releasing the towel, the toes are extended and abducted. Although we speculated that the abductor hallucis longus muscle was used, we observed that the extensor muscle group contracted at the same time. Previous studies using EMG focused only on the flexor muscle group because of its characteristics, which are yet to be clarified^10^.

The present results indicate that foot rock-paper-scissors is effective for exercising the medial longitudinal arch, while towel gathering is effective for improving the toe grip force. Furthermore, Kelly et al.^28^ revealed a positive correlation between the activities of the abductor hallucis, flexor digitorum brevis, and quadratus plantae. These muscles which are crucial for postural control, and are recruited in a highly coordinated manner for stabilizing the foot and maintaining balance in the mediolateral direction, particularly during single-leg positions. This suggests that continued training with towel gathering may also be effective in reducing the sway due to the center of gravity.

This study had several limitations. First, the FDG-PET method captures only the muscle glucose uptake. Although other substrates such as free fatty acids, muscle glycogen, and lactate are also metabolized in active myocytes, glucose oxidation increases with exercise intensity, and glucose uptake increases in proportion to glycogen utilization when exercise intensity rises^17^. In addition, previous reports have shown that FDG uptake is higher in muscles composed of type I fibers than in muscles composed of type II fibers^29^. Therefore, this result may not completely reflect all skeletal muscle activity. Second, the study included only a single session of training and experimentation, preventing us from examining continuous effects. Future studies should aim to evaluate the effects of continuous training sessions on skeletal muscle activity, changes in toe muscle strength, and improvements in static balance ability. Indeed, the comprehensive patterns of skeletal muscle activity during commonly performed foot exercises are unknown, and the skeletal muscles related to the control of static balance and extent of their contributions are yet to be clarified. Third, since a manual method was used to measure the SUV, the ROI range may not be accurate. Fourth, the measurement was performed in one slice using the landmark as an index, meaning that it does not reflect the activity of the entire muscle. However, data from our previous studies suggest that the difference is not significant^22,26^. Fifth, because of the low spatial resolution of PET, it needs to be combined with another imaging modality that can more accurately spatially identify lesions that show abnormal high absorption on PET. Typically, PET is combined with CT to provide this anatomical map. Since the PET/CT system used in this study used time of flight, it was impossible to obtain a higher spatial resolution, which is a limitation of PET cameras. In order to optimally assess the role of PET in various situations, it is necessary to take into account a phenomenon known as partial volume effect (PVE)^30^, which is greater in areas with low FDG accumulation than in areas with high accumulation. However, we evaluated areas of high FDG accumulation in this study, and we believe that the effect of PVE is not as great as in areas of low FDG accumulation. Lastly, this study was conducted on trained athletes. The results of this study may not be generalizable to patients with lower extremity disorders.

## Conclusion

This is the first study to apply FDG-PET to comprehensively investigate lower-limb skeletal muscle activity during foot exercise, despite the aforementioned limitations. Given that the foot rock-paper-scissors and towel gathering exercises target different skeletal muscles, the two techniques should be taught and implemented according to their respective purposes.

## Data Availability

The datasets generated and/or analyzed during the current study are not publicly available due to further analysis of data for upcoming publications, but are available from the corresponding author on reasonable request.
